# Vaccination With the Commensal *Streptococcus mitis* Expressing Pneumococcal Serotype 5 Capsule Elicits IgG/IgA and Th17 Responses Against *Streptococcus pneumoniae*

**DOI:** 10.3389/fimmu.2021.676488

**Published:** 2021-04-19

**Authors:** Sudhanshu Shekhar, Heidi A. Åmdal, Fernanda Cristina Petersen

**Affiliations:** Institute of Oral Biology, University of Oslo, Oslo, Norway

**Keywords:** *Streptococcus mitis*, Immunity, Antibody, Th17, *Streptococcus pneumoniae*

## Abstract

Recent studies have identified a clinical isolate of the commensal *Streptococcus mitis* that expresses *Streptococcus pneumoniae* serotype 5 capsule (*S. mitis* serotype 5) and shows serospecificity toward pneumococcal serotype 5. However, it remains unknown whether *S. mitis* serotype 5 induces protective immunity against pneumococcal serotype 5. In this study, we evaluated the ability of *S. mitis* serotype 5 to generate protective immunity in a mouse model of lung infection with pneumococcal serotype 5. Upon challenge infection with *S. pneumoniae* serotype 5, mice intranasally immunized with *S. mitis* serotype 5 exhibited reduced pneumococcal loads in the lungs, nasal wash, and bronchoalveolar lavage fluid compared with those receiving PBS (control). The immunized mice displayed significantly higher levels of IgG and IgA antibodies reactive to *S. mitis* serotype 5, *S. pneumoniae* serotype 5 or *S. pneumoniae* serotype 4 than the antibody levels in control mice. In vaccinated mice, the IgG/IgA antibody levels reactive to *S. mitis* serotype 5 or S. *pneumoniae* serotype 5 were higher than the levels reactive to *S. pneumoniae* serotype 4. Furthermore, *in-vitro* restimulation of the lung-draining mediastinal lymph node cells and splenocytes from immunized mice with killed *S. mitis* serotype 5, *S. pneumoniae* serotype 5 or *S. pneumoniae* serotype 4 showed enhanced Th17, but not Th1 and Th2, responses. Overall, our findings show that mucosal immunization with *S. mitis* serotype 5 protects against *S. pneumoniae* serotype 5 infection and induces Th17 and predominant serotype-specific IgG/IgA antibody responses against pneumococcal infection.

## Introduction

*Streptococcus pneumoniae* is an important human pathogen that causes a range of diseases, including sepsis, meningitis, and pneumonia, and poses a threat to public health worldwide (1, 2). According to the World Health Organization, *S. pneumoniae* is responsible for approximately 1.6 million deaths per annum, particularly among young children  (1, 3). *S. pneumoniae*’s capsular polysaccharides are considered to be the most important virulence factor by protecting pneumococci from immune cell-mediated phagocytosis. A pneumococcal serotype is characterized by serological reactivity to and molecular structure of its capsular polysaccharides. The immunity ensued against the capsular serotype is protective and has crucial implications for vaccine development (4–6). More than 90 serotypes have thus far been reported (4). Out of these pneumococcal serotypes, serotype 5 is strongly associated with invasive pneumococcal disease (IPD), with an invasiveness index that is 60 times higher than those of some of the least invasive serotypes (7, 8). And so, this serotype is contained in pneumococcal conjugate vaccine (PCV) and pneumococcal polysaccharide vaccine (PPSV) formulations.

*Streptococcus mitis*, a commensal bacterium, colonizes the mucosal surfaces of the human oral cavity and upper respiratory tract, and shares a major proportion of its genome with *S. pneumoniae* (9, 10). Our previous studies using humans and animal models have reported that antibody (IgG/IgA) and T helper cell (Th) 17 (Th17) responses specific for *S. mitis* show cross-reactivity with pneumococcal serotypes (11–13). Intranasal immunization of mice with live *S. mitis* triggered serotype-independent immunity against pneumococcal lung infection (13). Upon vaccination with genetically engineered *S. mitis* that expresses pneumococcal serotype 4 capsule (*S. mitis* TIGR4cps), mice generated enhanced protection against *S. pneumoniae* serotype 4 in a serotype-dependent fashion (13). Recently, Pimenta *et al.* have recovered commensal isolates of mitis streptococci from the upper respiratory tract of adult individuals that were PCR-positive for the pneumococcal serotype 5 specific gene (*wzy*5) (14). The *S. mitis *(hereinafter called as *S. mitis* serotype 5) shared the highest similar capsular polysaccharide biosynthetic gene cluster (*cps*5) with the same order in pneumococcal serotype 5 (14). Furthermore, antisera specific for *S. mitis* serotype 5 showed reactivity with pneumococcal serotype 5, indicating a positive Quellung reaction, and induced serotype 5-specific opsonophagocytosis (14).

In the present study, we specifically aimed to investigate whether *S. mitis* serotype 5 confers protective immunity against *S. pneumoniae* using a mouse model of pneumococcal lung infection. We also assessed the associated adaptive immune (IgG/IgA and Th) responses ensued due to immunization with *S. mitis* serotype 5. Our findings from this study provide important insights into how the naturally occurring commensal *S. mitis* that expresses pneumococcal serotype 5 can be used to generate protective immunity against infections with *S. pneumoniae*, which may have implications for the development of vaccines that contain serotypes that are prevalent in a particular geographical area.

## Materials and Methods

### Bacterial Strains and Media

*S. mitis* serotype 5 (KE67013) was kindly provided by the CDC, Atlanta, USA (14). *S. pneumoniae* serotypes included were *S. pneumoniae* serotype 5 (ATCC 6305; CCUG 33774) and *S. pneumoniae* serotype 4 (TIGR4). The bacterial strains were suspended in trypticase soy broth (Becton Dickinson, Franklin Lakes, NJ, USA) and 15% glycerol and stored in −80°C freezer. For the use of bacteria, stock cultures were diluted and grown at 37°C to an optical density (OD) of 0.5 at 600 nm in a 5% CO_2_ incubator. The bacterial cells were harvested by centrifugation at 5,000 *g* for 10 min at 4°C and washed in endotoxin free Dulbecco’s-PBS (Sigma-Aldrich, St. Louis, MO, USA).

### Mice

Swiss mice used in this study were females of 6-8 weeks age. These mice were specific pathogen free (SPF) and bought from the JANVIER LABS, France, and quarantined and housed in a Minimal Disease Unit at the animal facility at Oslo University Hospital, Rikshospitalet, Oslo, Norway. The mice were kept in isocages that are environmentally enriched with impellers and paper nest building, and given standard feed and water ad libitum. All mouse experiments were approved by the Norwegian Food Safety Authority, Oslo, Norway (Project license number FOTS – 22302) and performed in accordance with the guidelines of the Norwegian Animal Welfare Act (10 June 2009 no. 97), the Norwegian Regulation on Animal Experimentation (REG 2015-06-18-761) and the European Directive 2010/63/EU on the Protection of Animals used for Scientific Purposes. Mice were allowed a one week acclimatization period before experiments were started.

### Immunization and Challenge Infection

To perform immunization, mice were anesthetized with isoflurane (4%), followed by intranasal administration of 5 x 10^7^ colony forming units (CFU) of *S. mitis serotype 5* in 20 µl of PBS or 20 µl of PBS (control) for each mouse at days 0, 14, and 21. The immunized mice were anesthetized with isoflurane (4%) at 24 hours after the last immunization, followed by intranasal instillation with 8 x 10^6^ CFU of *S. pneumoniae* serotype 5 suspended in 50 µl of PBS, as described previously (13). Of note, we performed our experiment with 4 mice in immunized and 4 mice in control group, and the experiment was repeated to confirm the findings. The data represented in figures are pooled from the results of these two independent experiments.

### Sample Collection

Mice were euthanized at 24 hours after pneumococcal challenge, and the nasal wash, bronchoalveolar lavage fluid (BALF), spleen, blood, lungs, and lung-draining mediastinal lymph nodes were collected and stored in ice for further processing. For euthanasia, mice were anesthetized with isoflurane (4%) and then inoculated with an intraperitoneal injection of pentobarbital (0.5 ml per mouse). To obtain antisera, the freshly isolated blood was kept at 4°C for 1 hour and then centrifuged at 1000g for 5 minutes. The supernatant antisera were collected and preserved at -80°C freezer for analysis. The nasal wash, BALF, and lungs were collected from the euthanized mice, as described previously (15). To recover the BALF and nasal wash, a small cut in the trachea was made with a scissor and 1 ml of sterile cold PBS was inoculated with a syringe (19 gauge needle) and recovered for plating as well as antibody measurements. The lungs were mashed on a 70 µm cell strainer (ThermoFisher Scientific, Rockford, IL, USA) with the plunger of a 3 ml syringe and washed with 1 ml PBS for CFU counting and cytokine analysis. The nasal wash, BALF, and lung samples were plated onto blood agar plates containing gentamicin (5 µg·ml^−1^) for differentiation from other species and CFU calculation (13, 15).

### Measurement of Antibody Responses

To determine antibody levels in mouse samples, a whole cell ELISA was used as described previously (12, 13). In brief, each well of a 96-well plate (Maxisorb, Nunc, Thermo Scientific) was coated overnight with 100 µl of bacterial suspension (OD_600_ 0.5), which was washed and then fixed with 10% formalin. The plate was washed and blocked with a blocking buffer (PBS + 0.05% Tween + 1% BSA) and incubation for 1 h at 37°C. Sera (1:1000), BALF (1:10) and nasal wash (1:10) were diluted, and added to wells in duplicate, incubated for 2 hours at room temperature before addition of the anti-IgG/HRP or anti-IgA/HRP secondary antibody (1:10000) followed by incubation for 2 h at room temperature. The plates were washed and 100 µl of TMB substrate (ThermoFisher Scientific, Rockford, IL, USA) was added to each well. The plates were incubated in the dark at room temperature for 15 min, after which stop solution (ThermoFisher Scientific, Rockford, IL, USA) was added to each well to terminate the reaction. Absorbance was measured by reading the plates at 450 nm using a Multi-Mode reader (BioTek™ Cytation™ 3; ThermoFischer Scientific).

### Cell Isolation From the Lymph Nodes and Spleen

The mediastinal lymph nodes and spleen were removed from the euthanized mice and processed into single‐cell suspensions. The mediastinal lymph nodes and spleen were mashed on a 70 µm cell strainer (ThermoFisher Scientific, Rockford, IL, USA) with the plunger of a 3 ml syringe and washed with the washing buffer (PBS, 0.5% BSA and 5 mM EDTA). The cell suspension was lysed with RBC lysis buffer and washed twice. Hemocytometer and trypan blue were used to count viable cells.

### *In Vitro* Restimulation of the Lymph Node Cells and Splenocytes

5 x 10^6^ splenocytes in one ml or 1 x 10^6^ lymph node cells in 200μl of complete RPMI medium (10% heat‐inactivated FBS, 25 μg/mL gentamicin, L‐glutamine, and sodium bicarbonate; Sigma-Aldrich, UK) were cultured at 37°C, and restimulated with UV-killed *S. mitis* serotype 5, *S. pneumoniae* serotype 5 or *S. pneumoniae* serotype 4 (10^5^ CFU/ml) for 72 hours. The culture supernatants were frozen at -80^0^C for further cytokine analysis by commercial ELISA kits. The concentrations of IFN-γ, IL-4, and IL-17A were measured by Ready-SET-Go ELISA kits (eBioscience, San Diego, CA, USA), whereas the concentration of IL-4 was measured with an ELISA kit from Invitrogen (Vienna, Austria), in accordance with the instructions of respective manufacturers. The cytokine detection limit of the ELISA kits for IL-17, IL-4, and IFN-γ were 4, 3.9, and 15 pg/ml, respectively. Similarly, the cytokine levels in the lung homogenates, nasal wash and BALF were analyzed.

### Statistics

Unpaired Student’s *t* test was used for comparing two groups (GraphPad Prism Software, version 7, Graph Pad, San Diego, CA, USA). A *p* value less than 0.05 was considered significant.

## Results

### Intranasal Immunization of Mice With *S. mitis* Serotype 5 Protects Against Challenge Infection With *S. pneumoniae* Serotype 5

**Figure 1:**
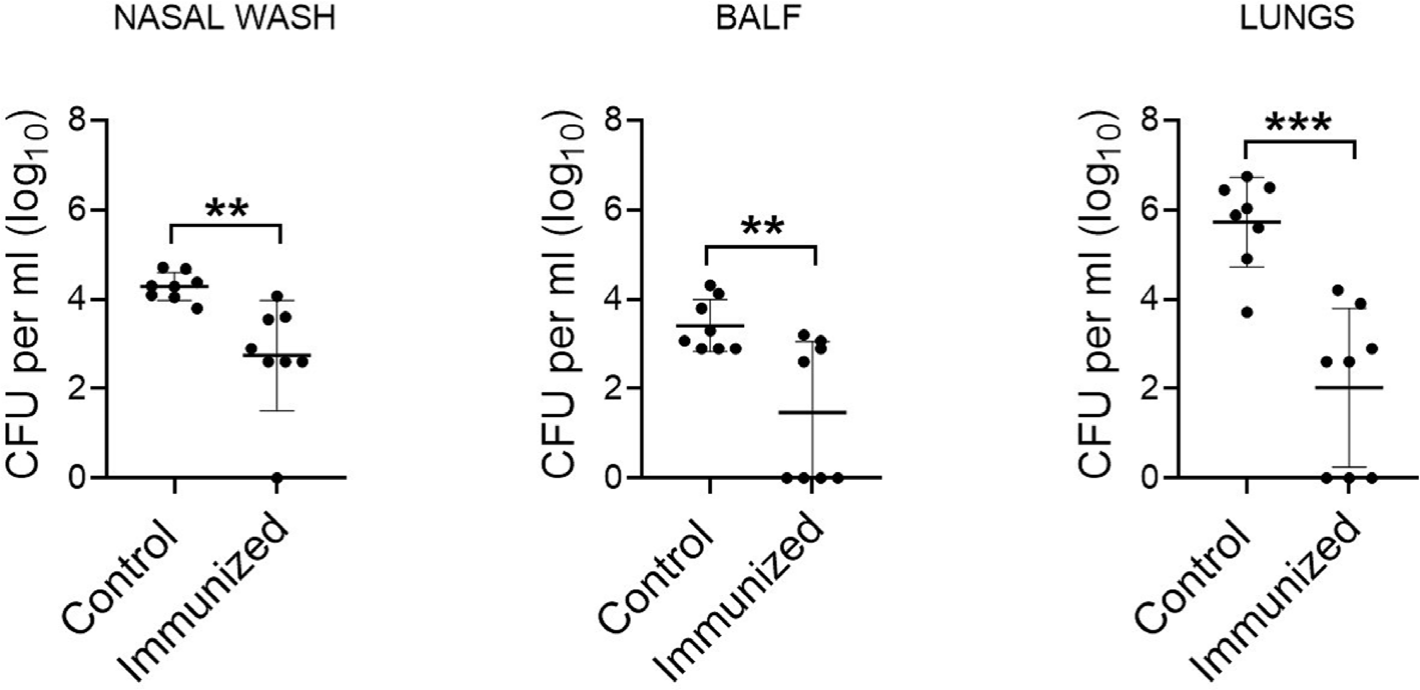
Protective efficacy of *S. mitis* serotype 5 against lung infection with pneumococcal serotype 5. Mice were intranasally immunized with *S. mitis* serotype 5 or PBS (control) at days 0, 14, and 21, and then subjected to lung infection with *S. pneumoniae* serotype 5. They were sacrificed at 24 hours following the challenge infection, and nasal wash, BALF, and lungs were collected for the analysis of pneumococcal load (CFU). Data are shown as mean ± SD and pooled from the results of two independent experiments with 4 mice in each group. Each symbol represents data from an individual mouse, and the horizontal bars are mean values of the groups. **p<0.01; ***p<0.001. Unpaired Student’s *t* test.

Accumulating evidence has shown that *S. mitis* serotype 5 exhibits antigenic relatedness to pneumococcal serotype 5 (14). To assess the protective efficacy of *S. mitis* serotype 5, we intranasally immunized mice with live *S. mitis* serotype 5 thrice followed by lung infection with *S. pneumoniae* serotype 5. The immunized mice exhibited significantly reduced pneumococcal loads in the nasal wash, BALF, and lungs compared to the control mice receiving PBS (**Figure 1**). Of note, *S. mitis* serotype 5 immunization completely eliminated pneumococcal burden from the BALF of 50% of all the mice immunized, whereas none of the control mice was able to totally clear pneumococcal loads from any of the samples collected. Thus, *S. mitis* serotype 5 confers strong protection against lung infection with *S. pneumoniae* serotype 5. Of note, a table showing CFU counts from each mouse used in this study has been included (**Supplementary Table 1**).

### *S. mitis* Serotype 5 Induces Predominant Serotype-Specific IgG and IgA Antibody Responses

**Figure 2:**
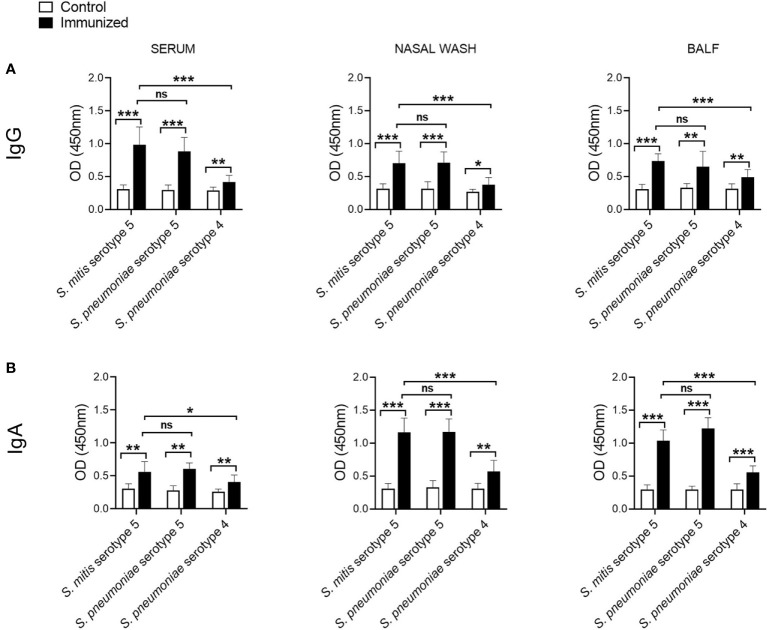
IgG and IgA responses to *S*. *pneumoniae* following immunization with *S. mitis* serotype 5. Mice were immunized with *S. mitis*, which was followed by pneumococcal lung infection, and the nasal wash, BALF and sera samples were collected to analyze **(A)** IgG and **(B)** IgA responses. Levels of IgG and IgA antibodies reactive to pneumococcal serotypes in the sera were measured by whole cell ELISA. The sera were diluted 1:1000, and the nasal wash and BALF 1:10. Data are shown as mean ± SD and pooled from the results of two independent experiments with 4 mice in each group. *p<0.05; **p<0.01; ***p<0.001. Unpaired student’s *t* test. ns, not significant.

Our recent findings demonstrate that IgG and IgA antibodies from mice immunized with the *S. mitis* type strain, which has a capsule locus not found in *S. pneumoniae*, reveal reactivity with *S. pneumoniae* serotypes 2 and 4, suggesting an antibody-mediated response that is independent of the pneumococcal serotype (13). It is further shown that antisera raised against *S. mitis* serotype 5 show serotype-specific reactivity with *S. pneumoniae* serotype 5 (14). To have detailed knowledge on how IgG and IgA antibodies specific for *S. mitis* serotype 5 react with different pneumococcal serotypes, we immunized mice with *S. mitis* serotype 5, which was followed by lung infection with *S. pneumoniae* serotype 5, and examined IgG and IgA responses in the nasal wash, BALF, and sera using a whole cell ELISA. The mice immunized with *S. mitis* serotype 5 displayed significantly higher levels of IgG antibodies binding to *S. mitis* serotype 5, *S. pneumoniae* 4 or *S. pneumoniae* 5 compared with IgG levels in control mice (**Figure 2A**). However, IgG levels in immunized mice reactive to *S. pneumoniae* 4 were much lower than the IgG levels binding to *S. mitis* serotype 5 or *S. pneumoniae* 5 (**Figure 2A**). In accordance with these findings, the immunized mice exhibited increased binding of IgA antibodies to *S. pneumoniae* 5 or *S. pneumoniae* 4 compared with control mice (**Figure 2B**). And, IgA levels binding to *S. mitis* serotype 5 or *S. pneumoniae* 5 were significantly higher than the levels reactive to *S. pneumoniae* 4 (**Figure 2B**). Cumulatively, intranasal immunization with *S. mitis* serotype 5 induces mucosal and systemic IgG and IgA antibody responses to pneumococcal serotype 5 and 4, although the response in case of serotype 5 is stronger than serotype 4, suggesting a predominant serotype-specific bias.

### *S. mitis* Serotype 5 Generates Enhanced Th17 Responses to Pneumococcal Infection

**Figure 3:**
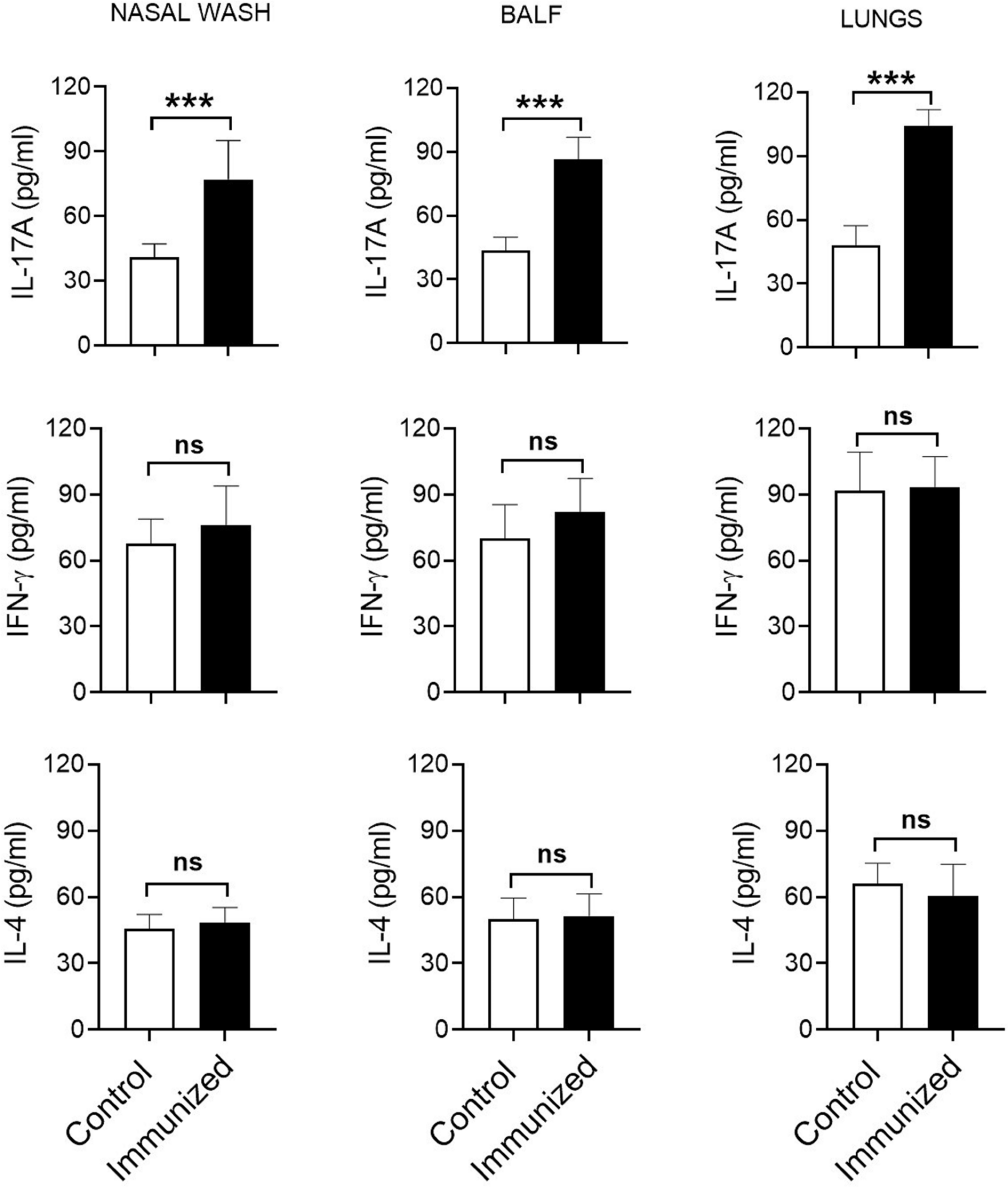
Cytokine responses in the respiratory tract after immunization. Nasal wash, BALF, and lungs were collected from the immunized mice subjected to pneumococcal challenge infection, and the cytokine levels were measured using ELISA. Data are shown as mean ± SD and pooled from the results of two independent experiments with 4 mice in each group. ***p<0.001. Unpaired student’s *t* test. ns, not significant.

**Figure 4:**
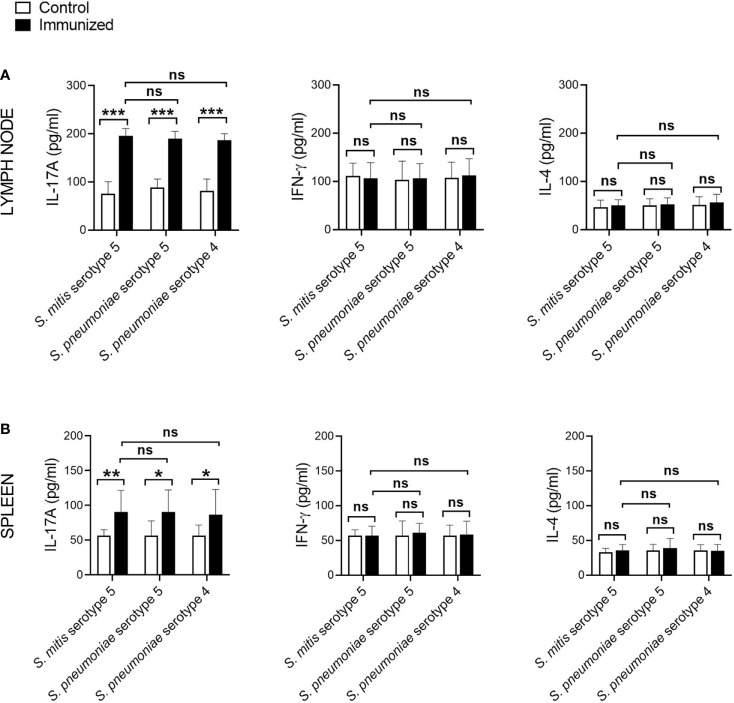
Antigen-specific cytokine production by the lymph node cells and splenocytes following immunization. The mediastinal lymph node cells and splenocytes from the *S. mitis* serotype 5-immunized or control mice subjected to pneumococcal challenge were restimulated with killed *S. mitis* serotype 5, *S. pneumoniae* 5 or *S. pneumoniae* 4 for 72 hours, and the cytokine levels (IL-17A, IFN-γ, and IL-4) in the culture of **(A)** lymph node cells and **(B)** splenocytes were measured by ELISA. Data are shown as mean ± SD and pooled from the results of two independent experiments with 4 mice in each group. *p<0.05; **p<0.01; ***p<0.001. Unpaired student’s *t* test. ns, not significant.

Th, particularly Th1 and Th17, responses play a crucial role in protective immunity to pneumococcal lung infections (16, 17). We have previously reported that mucosal vaccination with *S. mitis* triggers local IL-17A/Th17 immunity in the respiratory tract of mice (13). In the present study, we used *S. mitis* serotype 5 to assess its ability to generate major T helper cell responses – Th1, Th2, and Th17 – at the mucosal and peripheral tissues. First, we examined the cytokine profile of local tissues following immunization of mice with *S. mitis* serotype 5 followed by lung infection with pneumococcal serotype 5. The immunized mice produced large quantities of IL-17A (Th17) in the nasal wash, BALF, and lungs compared with mice receiving PBS (**Figure 3**). However, IFN-γ (Th1) and IL-4 (Th2) cytokine levels were not statistically different between the immunized and control groups (**Figure 3**). In order to acquire knowledge on antigen-specific T cell responses, we restimulated the mediastinal lymph node cells from the immunized mice with killed *S. mitis* serotype 5, *S. pneumoniae* 5 or *S. pneumoniae* 4 and measured the cytokine responses. The lymph nodes from immunized mice produced higher levels of Th17, but not Th1 and Th2, cytokines compared with control mice (**Figure 4A**). In immunized mice, the lymph nodes cells restimulated with *S. mitis* serotype 5, *S. pneumoniae* 5 or *S. pneumoniae* 4 secreted similar levels of Th17 cytokines, which were higher than in the control mice (**Figure 4A**). Then, we investigated the antigen-specific cytokine production pattern at the peripheral tissues (spleen) in response to restimulation with *S. mitis* serotype 5, *S. pneumoniae* 5 or *S. pneumoniae* 4. In line with the findings from the lymph node cell restimulation (**Figure 4A**), we found that Th17, but not Th1 and Th2, cytokine levels produced by the splenocytes restimulated with *S. mitis* serotype 5, *S. pneumoniae* 5 or *S. pneumoniae* 4 were significantly increased compared with the cytokine levels in control mice (**Figure 4B**). Thus, these findings indicate that mucosal vaccination with *S. mitis* serotype 5 induces Th17 immunity both at the local and peripheral levels.

## Discussion

The rare occurrence of serotype 5 invasive disease in the United States and Kenya has led to the hypothesis that the expression of serotype 5 capsule by non-pneumococcal Mitis group streptococci, including *S. mitis*, may confer cross-species immunity (18–20). Our present study supports this hypothesis because intranasal immunization of mice with *S. mitis* serotype 5 triggered protective immunity against nasal colonization by and lung infection with *S. pneumoniae* serotype 5. Our data show that the mice immunized with *S. mitis* serotype 5 exhibited significantly lower pneumococcal loads in the nasal wash, BALF, and lungs following challenge with *S. pneumoniae* 5. Of note, the pneumococcal loads in the immunized mice were around 2-3 times lower in the BALF and lungs, and 50% of the immunized ones had nil pneumococcal CFU in the BALF. This protective efficacy is higher than what has been demonstrated in similar mouse models where mice intranasally inoculated with *S. pneumoniae* serotype 6 (BHN418 6B) received a challenge with homologous pneumococci (21). Furthermore, *S. mitis* serotype 5 elicited robust IgG and IgA immune responses locally and systemically, which were predominantly directed against the serotype 5 capsule. It is however important to mention that *S. mitis* serotype 5 also elicited IgG/IgA antibody levels that were reactive to *S. pneumoniae* serotype 4, suggesting a serotype-independent immune response. Recently, we showed that intranasal immunization of mice with live *S. mitis* induces serotype-independent protection against infections with pneumococcal serotypes 2 and 4, which was associated with enhanced IgG and IgA responses (13). Likewise, vaccination with attenuated *S. pneumoniae* serotype 4 provided serotype-independent protection against invasive pneumococcal infections (22). The reason behind this serotype-independent immune response could be explained by the similarity between the nature of capsular and protein antigens of *S. mitis* and *S. pneumoniae*. In addition to immunogenic capsular antigens, *S. pneumoniae* possesses certain protein antigens, such as choline-binding protein D (CbpD), which are highly conserved between *S. mitis* and *S. pneumoniae* (12). Thus, the antibody responses ensued due to *S. mitis* serotype 5 in this study may be generated against capsular and protein antigens of *S. pneumoniae* serotype 5, and protein antigens of *S. pneumoniae* serotype 4.

Our current study shows that *S. mitis* serotype 5 vaccination exerts an increased antigen-specific IL-17A/Th17 immune response at the mucosal (respiratory tract) and peripheral (spleen) tissues, which is critical for protective immunity to pneumococcal lung infections (15). However, the IL-17A/Th17 responses against *S. pneumoniae* serotype 5 and *S. pneumoniae* 4 were similar, suggesting that these responses are induced against shared protein antigens between *S. mitis* serotype 5 and *S. pneumoniae* serotypes 4 and 5. This is in accordance with our previous studies where *S. mitis*-specific human Th17 cells showed cross-reactivity with *S. pneumoniae* (11), and that mice immunized with *S. mitis* promoted Th17 immunity (13). However, it remains to be ascertained whether *S. mitis* serotype 5-induced IL-17A/Th17 immunity plays a direct role in protection against pneumococcal infection, which will be addressed in our future studies by using IL-17 knockout mice. It is notable here that although Th responses, especially Th1, contribute to host defense against pneumococcal lung infection (16, 17), we failed to see any difference in Th1 (IFN-γ) and Th2 (IL-4) cytokine levels between the immunized and control groups at 24 hours after the last *S. mitis* serotype 5 immunization. Similar observations were found in our previous study where the Th response was assessed at day 7 after immunization with the *S. mitis* type strain (13). Moreover, a previous study showed that prior nasopharyngeal colonization of mice by *S. pneumoniae* resulted in significant increases in the BALF levels of Th1 cytokine (TNF-α) at 4 hours, but not at 18 hours, following pneumococcal challenge (23). Therefore, in the present study, the reason as to why we did not find difference in Th1/Th2 cytokine levels could be attributed to the time point (24 hours post-immunization) chosen to analyze the cytokines. Future studies need to assess the cytokine levels at various time points following immunization.

It is also important to discuss how relevant our findings from a mouse model are for evaluation of human-commensal effects as many gut commensals induce tolerance in the natural host (24). Previous studies in humans have shown that there exists a humoral cross-reactivity between *S. pneumoniae* and *S. mitis* (12, 25, 26), and that intranasal inoculation of adult humans with the oral streptococcal commensals induce protective immunity against otitis media caused by bacterial pathogens, including *S. pneumoniae* (27). Furthermore, inoculation with the nasopharyngeal commensal *Neisseria lactamica* in healthy individuals intranasally elicits cross-reactive systemic opsonophagocytic antibodies to the pathogen *Neisseria meningitidis* (28). Thus, these human studies indicate an important role for the oral/nasopharyngeal commensals in inducing protective immunity against respiratory pathogens. In the times to come, we plan to perform a study to investigate the impact of *S. mitis* colonization/immunization on incidence of pneumococcal carriage/infection.

In conclusion, our present study demonstrates that mucosal vaccination with live *S. mitis* serotype 5 protects against infection by *S. pneumoniae* serotype 5, and induces strong IgG/IgA and Th17 responses against pneumococcal infection at the local and systemic/peripheral tissues. Moreover, the IgG/IgA antibody levels reactive to *S. mitis* serotype 5 or *S. pneumoniae* serotype 5 were higher than the levels reactive to *S. pneumoniae* serotype 4, indicating a serotype-specific response. These findings provide significant insights into how naturally occurring commensal streptococci that express pneumococcal serotype capsule can be exploited to generate protective immunity against pneumococcal infections.

## Data Availability Statement

The original contributions presented in the study are included in the article/**Supplementary Material**. Further inquiries can be directed to the corresponding authors.

## Ethics Statement

The animal study was reviewed and approved by Norwegian Food Safety Authority, Oslo, Norway (Project license number FOTS – 22302).

## Author Contributions

SS designed research studies, conducted experiments, acquired and analyzed data, and wrote the paper. HÅ conducted experiments, acquired, and analyzed data. FP designed research studies, analyzed data, and wrote the paper. All authors contributed to the article and approved the submitted version.

## Funding 

We thank the Norwegian Research Council (grant numbers – 273833 and 274867) for financial support.

## Conflict of Interest

The authors declare that the research was conducted in the absence of any commercial or financial relationships that could be construed as a potential conflict of interest.
