# Supplementary material for: Vaccination With the Commensal Streptococcus mitis Expressing Pneumococcal Serotype 5 Capsule Elicits IgG/IgA and Th17 Responses Against Streptococcus pneumoniae
Source: Front Immunol. 2021 Apr 19;12:676488. doi: 10.3389/fimmu.2021.676488 (PMC8089380; doi:10.3389/fimmu.2021.676488)
Supplement: Supplementary file 1 [file DataSheet_1.pdf]

EXPERIMENT – 1

IMMUNIZED MICE (n=4)	NASAL WASH (CFU/ml)	BALF (CFU/ml)	LUNGS (CFU/ml)
1	400	0	800
2	400	800	8000
3	400	0	0
4	12000	400	400
**CONTROL MICE (n=4)**			
1	48000	2000	3200000
2	52000	800	5200
3	19200	20800	400000
4	20000	800	2800000

EXPERIMENT – 2

IMMUNIZED MICE (n=4)	NASAL WASH (CFU/ml)	BALF (CFU/ml)	LUNGS (CFU/ml)
1	800	1200	16000
2	3600	1600	400
3	4000	0	0
4	0	0	0
**CONTROL MICE (n=4)**			
1	12400	1200	80000
2	11200	6400	1080000
3	6400	800	760000
4	24000	13200	5600000

Supplementary Table 1: Pneumococcal load in mice immunized with *Streptococcus mitis* expressing pneumococcal serotype 5 capsule (*S. mitis* serotype 5) following infection with *Streptococcus pneumoniae* serotype 5. Mice were intranasally inoculated with *S. mitis* serotype 5 (immunized) or PBS (control) at days 0, 14, and 21, and then subjected to lung infection with *S. pneumoniae* serotype 5. They were sacrificed at 24 hours following the challenge infection, and nasal wash, bronchoalveolar lavage fluid (BALF), and lungs were collected for the analysis of pneumococcal load in terms of colony forming units (CFU) per ml PBS. The first experiment (Experiment – 1) was done using 4 mice in each group, i.e. immunized and control groups (4+4 = 8), which was repeated with the same number of mice (Experiment – 2). This table shows the CFU counts per ml from both the experiments (1 and 2) that were performed independently.
